# Rethinking the occurrence of volunteering from the capability-opportunity-motivation perspective

**DOI:** 10.3389/fpsyg.2026.1799101

**Published:** 2026-04-07

**Authors:** Yijing Huang, Zhanni Luo, Kangzhou Peng

**Affiliations:** School of Foreign Languages and Literatures, Chongqing Normal University, Chongqing, China

**Keywords:** behavior change, behavioral change wheel (BCW), COM-B, volunteer motivation, volunteering

## Abstract

The COM-B model, proposed by Michie and colleagues, posits that capability (C), opportunity (O), and motivation (M) interact to generate behavior (B), offering a comprehensive framework for analyzing the conditions under which behavior occurs. While the COM-B model has been predominantly applied to health behavior interventions, we propose that it holds considerable potential for extension to positive behaviors such as volunteering. We introduce the COM-B framework to reconceptualize the occurrence of volunteering behavior, using university student volunteering as an illustrative case. We delineate the specific meanings of Capability, Opportunity, and Motivation within the volunteering context and explore conditions that enable volunteering as a behavior to occur. Regarding the Capability component, we distinguish between physical and psychological capability, emphasizing the central role of the latter for university student volunteering; Regarding opportunity, we differentiate physical opportunity from social opportunity and underscore the importance of perceived opportunity availability; Regarding the Motivation component, we integrate reflective and automatic motivation, proposing “wanting” as the critical mechanism that bridges the intention-behavior gap. We aim to provide a more comprehensive analysis of the conditions that generate volunteering behavior from the perspective of Capability, Opportunity and Motivation.

## 1 Introduction

Over the past several decades, researchers across psychology, public health, sociology, and behavioral economics have developed a large number of frameworks aimed at explaining and predicting human behaviors. These frameworks have provided the theoretical foundation for countless interventions designed to address pressing social and health challenges, such as physical activity promotion, medication adherence, smoking cessation, and chronic disease management. Among them, the COM-B model has emerged as one of the most influential frameworks. Proposed by Michie et al. (2011), the model conceptualizes behavior (B) as the product of interactions among three components: capability (C), opportunity (O), and motivation (M). Its strength lies in shifting the analytical focus from isolated predictors to the systemic conditions that enable behavior to occur. In this perspective paper, we argue that the COM-B model holds significant potential beyond the remediation of problem behaviors and can be extended to the cultivation of positive and prosocial behaviors, especially volunteering.

Volunteering is a behavior of considerable developmental and social significance, contributing to students’ social connectedness, skill development, and mental health (Alganami and El Keshky, 2025; Lv et al., 2024). University students are among the most representative groups in volunteering, making this population particularly relevant for examining how volunteering behavior occurs. Existing research on students’ volunteering has predominantly focused on classifying and cultivating motivation, drawing primarily on frameworks such as the Theory of Planned Behavior (TPB) and Self-Determination Theory (SDT), which are essentially intention- or motivation-centered models. However, motivation alone cannot fully account for behavior occurrence. The occurrence of volunteering behavior demands not only stronger motivation than typical behaviors, but also corresponding capabilities and environmental support which can be demonstrated through COM-B model. And how motivation translates into behavior also warrants further examination. In this perspective paper, we introduce the COM-B model and argue that volunteering, as a typical prosocial behavior (Penner et al., 2005), should be understood as the result of dynamic interactions among capability, opportunity, and motivation.

By reconceptualizing the conditions that generate volunteering behavior within the COM-B model, we aim to clarify the concept of capability, opportunity, and motivation about positive behaviors such as volunteering, and provide a more comprehensive analysis of volunteering in the context of university students, articulating what enables volunteering behavior to occur across the three components.

## 2 Capability: what enables students to volunteer?

Capability is defined as the individual’ s physical capacity (e.g., skills, strength, and stamina) and psychological capacity (e.g., knowledge and cognitive reasoning) necessary to perform a target behavior (Michie et al., 2011). In simpler terms, the capability concept answers the question: “What enables students to volunteer?” Specifically, physical capability refers to the physiological skills, strength, or endurance required to perform a behavior. It constitutes the biological foundation for behavior performance; Psychological capability involves the knowledge, cognitive processes, and mental skills necessary for performing behavior, such as memory, attention, decision-making, and self-regulation.

In relevant research on health behavior interventions, physical capability is regarded as the foundational prerequisite for behavioral intervention, and physical capability limitations directly constrain behavioral options (Brown et al., 2024; Paterson et al., 2024). This phenomenon, however, shifts considerably in the context of university student volunteering. Unlike clinical patients or older adults for whom physical limitations constitute primary behavioral barriers, university students are typically at the peak of physical vitality with minimal health constraints. Meanwhile, most campus and community-based volunteering activities require only basic physical mobility and general health, which are prerequisites that the vast majority of students readily meet. Consequently, within the context of university volunteering, the emphasis of discussion about the Capability component shifts from physical to psychological capability, which varies among students and thus become the major factor of generating university student volunteering.

Drawing on the COM-B model and the distinctive nature of volunteering, we propose that psychological capability for university student volunteering should be reconceptualized around four interrelated elements: volunteering knowledge, empathic capacity, self-regulation skills and interpersonal communication skills.

Volunteering knowledge constitutes the cognitive starting point. Knowledge and information have been identified as key factors influencing volunteering behavior (Mao et al., 2021; Fait et al., 2023), and all subsequent engagement rests on this factor. If potential volunteers, e.g., first-year students still unfamiliar with the campus environment, lack relevant information about volunteering, objectively available opportunities become effectively inaccessible for them. At the macro level, students need a basic understanding of social issues and needs; at the micro level, they need to have procedural knowledge and skills related to volunteering, including background knowledge in specific service domains, familiarity with basic procedural elements, and cognitive sills for different volunteer types, for example, medical volunteering necessitates basic healthcare skills; educational volunteering requires teaching abilities.

However, relevant knowledge does not automatically translate into effective volunteering behavior. Volunteering is fundamentally an altruistic, prosocial behavior centered on responding to others’ needs and circumstances. The ability to understand and share others’ emotional states, namely empathic capacity, serves as an important individual difference variable predicting long-term volunteering behaviors (Chen and Su, 2025). This capacity enables student volunteers to genuinely understand service recipients’ needs, respond appropriately, and derive meaning from service experiences, with which volunteering behavior can emerge and be sustained over time.

The involvement of empathic capacity carries inherent emotional costs. Volunteering is actually a kind of emotional labor behavior (Miao et al., 2025), which requires volunteers to have self-regulation skills to deal with various emotional challenges, including frustration when efforts yield limited results, negative emotions arising from interpersonal conflicts or pressure from complex volunteering tasks. Without adequate self-regulation skills, students may experience emotional exhaustion or compassion fatigue and finally withdrawal midway (Li et al., 2022), explaining why some initially enthusiastic students ultimately fail to develop stable patterns of volunteering behavior.

Beyond cognitive skills, social skills also constitute an important component of psychological capability. Volunteering behavior requires students’ sustained interaction with service recipients, fellow volunteers, and organizational coordinators, underscoring the importance of interpersonal communication skill in social skills. This skill involves listening to and understanding service recipients’ needs, coordinating with team members, and effectively communicating needs and difficulties to organizers. Strong interpersonal communication skill proves particularly critical for volunteer activities requiring frequent direct interaction with service recipients (Siqueira et al., 2022), such as educational and medical assistance.

Among university students, most possess foundational knowledge and basic physical fitness, yet exhibit significant variation in psychological skills that vary from person to person. Such variation directly influences subjective judgments of personal competence, thereby affecting volunteering motivation and behavior (Bang et al., 2023). Importantly, these volunteering-related psychological capabilities can be developed through training and experiential accumulation. Indeed, volunteering itself represents an important pathway for cultivating these capabilities (Haski-Leventhal et al., 2020). This implies a bidirectional relationship between capability and behavior: capability serves as a prerequisite for volunteer engagement, while volunteering experience reciprocally promotes capability development.

## 3 Opportunity: what makes volunteering possible?

Opportunity encompasses all factors external to the individual that enable or prompt behavior, including characteristics of the physical environment and determinants at the social or community level (Michie et al., 2011). Opportunity addresses whether the environment allows the behavior, serving as an external enabler. It answers the question: “What makes volunteering possible?” and can be further categorized into two sub-components: physical opportunity and social opportunity.

Physical opportunity refers to all objective conditions and resources within the physical environment that facilitate or impede behavior occurrence. It includes tangible, observable external factors. In the relevant studies, physical opportunity has been mainly manifested as time availability (Van Kasteren et al., 2020), facility placement (Gale et al., 2024), neighborhood walkability, proximity to parks and fitness facilities, and availability of exercise equipment (Webb et al., 2022). Moreover, the rise of digital platforms has further expanded the scope of physical opportunity, e.g., smart monitoring applications and e-health platforms for disease management (Hepburn et al., 2025).

Social opportunity encompasses the intangible, interpersonal conditions shaped by social interactions, collective atmospheres, and cultural norms and that either facilitate or constrain behavior. Central to this category is social influence, which manifests through multiple channels: social norms (the prevailing behaviors of those around us), social support (encouragement and assistance from others), and observational learning (the modeling effect of role figures). In studies about health behavior, social opportunity is primarily manifested through social norms, family values, support from family members, and opinions of friends (Patterson et al., 2023; Xie et al., 2023; Chen et al., 2026).

Volunteering, as a behavior relying on physical environment, is similarly shaped by physical opportunity, including accessibility of volunteer organizations and information, distance of location, flexibility of time scheduling, resources support, and physical environment suitability. Within these elements, accessibility of volunteer organizations and distance of service location have identified as frequently cited factors influencing volunteer participation in recent studies on volunteering behavior (Mitchell and Clark, 2021; Armstrong et al., 2023). In addition, recent COM-B application studies have increasingly identified several factors related physical environment suitability, involving different environment conditions like scenery, weather conditions, safety and comfort, and accessibility or quality of digital facilities (Brown et al., 2024; Westrop et al., 2025). We argue that it also represents an important influence on volunteering behavior, especially for university students who emphasize the sense of experience. Favorable and comfortable service environment contribute to positive volunteer experiences, thereby affecting participation motivation and sustainability (Cho et al., 2023). These elements constitute the material prerequisites for volunteering to occur. In the absence of accessible volunteer organizations, adequate activity information, manageable time schedules, or conveniently located service sites, volunteering is less likely to occur.

However, we observe that for university student volunteering, although physical opportunity barriers exist, they do not represent the most critical limiting factors. Most universities have established relatively well-developed volunteer organization networks, information dissemination channels have become increasingly diversified, and volunteer opportunities on and around campus are generally sufficient. More notably, even when physical opportunity conditions are comparable, participation rates continue to vary substantially across different student groups. This observation prompts us to shift our analytical focus toward the social opportunity.

Compared with health behaviors, volunteering typically occurs within highly institutionalized settings and is characterized by strong organizational embeddedness. This renders the social opportunity component particularly salient for volunteering and potentially more predictive than physical opportunity. Accordingly, university students’ volunteering behavior is profoundly shaped by organizational and cultural contexts. Therefore, beyond typical factors such as social norms and interpersonal influence, organizational support constitutes another critical factor in facilitating volunteering behavior. Specifically, in the university student volunteering context, social opportunity encompasses social relationship networks (peers, family, organizations, institutions) and cultural atmospheres that shape volunteering participation, such as peer influence, volunteer organization support, and organizational climate.

University represents a critical period for student socialization, during which peer influence exerts a profound effect on shaping students’ volunteering behavior. In volunteering participation, friends and classmates may exert influence through multiple mechanisms: social learning (observing peers’ volunteering behavior), social comparison (comparing one’s participation level with peers), direct invitation (being invited by friends), and social support (receiving encouragement from peers) (Michie et al., 2014). This primarily concerns the attitudes and consensus regarding volunteering participation within students’ reference groups (dormitories, classes, student organizations). When surrounding peers actively participate in volunteering, individuals perceive social expectations for participation and potential social pressure for non-participation.

Organizational support for volunteering is reflected in recognition of volunteers’ value and attention to their needs (Dekel et al., 2022), including process support, emotional support, and institutional incentives. Different kinds of supports ensure that during volunteer service, students can not only promptly receive assistance and guidance, but also experience emotional care and respect. Most importantly, they are able to gain formal recognition and rewards, including volunteer credit certification, bonus points in comprehensive quality assessments, consideration in awards and honors, and service certificate issuance. Although these recognition mechanisms do not constitute monetary compensation, they provide social returns that can enhance volunteers’ sense of efficacy and belonging.

Besides, organizational climate, as part of organizational culture, determines the quality of interpersonal environments within volunteer organizations or teams. A warm, supportive, inclusive atmosphere with a sense of belonging enhances volunteers’ participation enjoyment and sustained willingness; conversely, an indifferent, competitive, or exclusionary climate may lead to participant attrition. For many students, sustained volunteering is more likely to be motivated by enjoying being part of the team (Wu and Xu, 2022).

Social opportunity is not merely an external condition for behavior occurrence but also directly shapes motivation itself. Social norms, in particular, influence motivation through both descriptive norms (what others actually do) and injunctive norms (what others expect one should do) (Cialdini and Goldstein, 2004). When students observe their peers actively engaging in volunteering, they not only gain a sense of behavior recognition but also develop the desire to participate. This transformation from external norms to intrinsic motivation explains why universities with strong volunteering cultures tend to exhibit higher student participation rates.

We believe that in the Opportunity component, physical and social opportunity operate synergistically, with their combined effects often exceeding the sum of their individual contributions. A volunteer program that is convenient to access (high physical opportunity) while friends are participating and institutional incentives exist (high social opportunity) demonstrates significantly greater attractiveness and retention than programs excelling in only one dimension. In addition, there is also the compensatory relationship between these two dimensions. Strong social opportunity can partially offset moderate deficiencies in physical opportunity. For instance, when a volunteer project is located in distant service area with inconvenient transportation, students may still actively participate if the volunteer team atmosphere is harmonious, peer relationships are close, and organizer support is strong (Traeger et al., 2023). Similarly, when activity schedules partially conflict with classes, physical barriers may be overcome if social support is sufficiently robust.

## 4 Motivation: what drives students to volunteer?

Motivation is defined broadly as the internal psychological processes that energize and direct behavior, comprising both reflective processes (e.g., goal-directed planning and evaluation) and automatic processes (e.g., emotions and habits), which corresponds to reflective motivation and automatic motivation. This concept answers the question: “What drives students to volunteer?” Understanding how these two motivational types operate in the volunteering context is essential for explaining why some students volunteer while others do not.

Reflective motivation emerges from deliberate, conscious processes of reasoning, planning, and evaluating. Reflective motivation is primarily manifested through behavioral evaluation, planning, and goal setting, specifically encompassing self-efficacy, confidence in positive behavioral outcomes, perceived benefits of the behavior, value judgments regarding the behavior, and identity (Shpendi et al., 2025). Among these, self-efficacy has been identified as a key factor influencing a wide range of behaviors (Willmott et al., 2021).

Automatic motivation arises from immediate emotions, desires, impulses, or habits. Compared with reflective motivation, it is a process that largely bypasses deliberate reflection and is instead driven mainly by affective states, momentary experiences, and habits (McClintic et al., 2022; Schwarz et al., 2024).

A large number of theoretical and applied research on volunteering motivation has accumulated, drawing on frameworks such as the functional approach, self-determination theory, and social exchange theory. These studies demonstrate that volunteering are typically driven by multiple, concurrent motivations, including value orientations such as altruism and social responsibility (Dong and Bavik, 2023), self-development goals including skill enhancement and career exploration (Zhou and Kodama Muscente, 2023), social needs such as interpersonal connection, identity affirmation, and belonging (Thoits, 2021), and emotional factors such as compassion and gratitude (Naqshbandi et al., 2023). Those related to evaluation, values, intentions and goal setting are reflective motivations. Others align more closely with automatic motivation, that is immediate emotional impulses triggered by witnessing others’ difficulties, or habitual patterns formed through sustained engagement over time.

In the context of encouraging university student volunteering engagement, we propose that, reflective motivation manifests primarily four forms: instrumental goals, value-driven motivation, role identity, and self-efficacy. Instrumental goals represent the most explicit form of motivation, oriented toward tangible benefits derived from volunteering, such as obtaining credits, scholarships, or certificates, accumulating practical experience, developing skills, or enhancing one’ s curriculum vitae; Value-driven motivation, particularly prosocial values, constitutes the moral dimension of reflective motivation. Students holding such motivation perceive helping others as right and meaningful, with their participation reflecting altruism and social responsibility; Role identity represents the most internalized form, as it implies that being a “volunteer” is integrated into students’ self-concept, with participation serving to affirm and reinforce this identity; Self-efficacy occupies a core and foundational position, referring to students’ confidence and belief about their ability to successfully finish volunteer tasks. Whether students believe they possess the capability to effectively help others and feel confident in handling challenges that may arise during service directly influences their willingness to participate. These motivations share the common characteristic of requiring conscious thought and evaluation.

Automatic motivation primarily comprises two categories: emotional drivers and habituation. Emotional drivers includes pre-service empathic impulse and positive emotional experience during service. Positive emotional experiences, e.g., the satisfaction of helping others, the feeling of being needed, and a sense of meaning, particularly serves as a significant predictor of continued volunteering engagement (Kifle Mekonen and Adarkwah, 2022); Habituation exists in a situation that volunteering behavior has become automatic or habitual. For long-term participants, automatically attending to volunteer activity announcements and registering whenever conditions permit has largely become a habitualized behavioral pattern, without requiring deliberate decision-making each time (Mullan et al., 2021).

However, there is a gap between students’ intentions and their execution of volunteering behavior, termed intention-behavior gap (Rhodes et al., 2022). With regard to engagement in university volunteering, many students possess the capability and opportunity, endorse the value of volunteering, and even express intentions to participate, yet never actually take action. West and Michie (2020) provide an insight for understanding and bridging this gap, proposing that motivation can only truly drive behavior when it is transformed into “wanting.” Accordingly, we argue that in the context of university student volunteering, “wanting” constitutes the core motivational force that propels volunteering behavior.

“Wanting” differs from other motivations in the intensity of willingness. Even if a student believe that helping others is important, or even feel obligated to contribute to society, volunteering only occurs when the desire to participate surpasses competing desires, e.g., resting, studying, or entertainment, at the moment of decision. In other words, whether behavior occurs depends on whether “wanting to volunteer” prevails over other competing wants at that critical moment. Within the COM-B model, “wanting” emerges from the synergistic interaction between reflective and automatic motivation. A bidirectional relationship exists between these two types: in a top-down manner, reflective-level recognition of volunteering value and benefits can activate corresponding emotional responses, making students more readily moved by service needs; in a bottom-up manner, strong emotional experiences and established habitual patterns can shape or even override rational evaluation, for instance, a student may respond to a peer’ s volunteer invitation without careful deliberation. When students believe at the reflective level that volunteering is valuable while simultaneously experiencing strong emotional attraction at the automatic level, “wanting” emerges and volunteering behavior follows.

Accordingly, we suggest that understanding the motivation component of university student volunteering should not remain at the level of categorizing different motivations, but attend to how these motivations converge into sufficiently strong “wanting” in specific contexts, thereby bridging the gap between intention and behavior. This also implies that promoting volunteering requires not only cultivating appropriate values but also creating service environments that elicit positive emotional experiences, allowing “wanting to participate” to emerge naturally at critical moments and translate into behavior.

## 5 Discussion

Regarding the capability component, although we noted earlier that physical capability does not constitute a primary factor for most university students and typical volunteering behaviors, this does not mean it can be entirely overlooked. Physical capability remains relevant to volunteer participation in two respects. First, certain specialized volunteer activities impose higher physiological demands, such as hiking to remote rural areas for educational outreach, manual labor in post-disaster reconstruction, or prolonged standing during large-scale events, all of which require adequate stamina and good health. In addition, certain volunteer activities require specific physical skills beyond basic mobility. Disaster relief volunteering necessitates the physical strength for debris clearance and heavy material transport; elderly care volunteering requires the stamina and coordination to assist with mobility transfers; and first aid volunteering demands the physical dexterity to perform procedures such as cardiopulmonary resuscitation or wound dressing (Carlton et al., 2022).

Besides, we can not ignore the fact that some university students nowadays experience sleep deprivation, irregular schedules, and lack of regular exercise. The resulting fatigue and diminished energy may serve as hidden obstacles to volunteering participation. Accordingly, youth health promotion initiatives at national and community levels may indirectly facilitate volunteering behavior by enhancing students’ overall physical condition, although not directly targeting volunteering. By contrast, psychological capability plays a more central role in university student volunteering. The development of this capability is profoundly shaped by socialization processes, and universities, as primary sites for student socialization, assume a leading role in cultivating psychological capability, for example, through curricular design, experiential education, and extracurricular activities that promote civic competence and prosocial skills (Haski-Leventhal et al., 2020). However, mature practical models for integrating the cultivation of these psychological capabilities into volunteering promotion strategies remain lacking. The design of specific measures and the evaluation of their effectiveness require further practical exploration and empirical investigation.

Regarding the opportunity component, we distinguish between physical and social opportunity, emphasizing the significant predictive role of the latter for volunteering behavior. Building on this distinction, we introduce the concept of perceived opportunity availability. The objective existence of physical opportunities does not means students’ subjective perception of them. A volunteer program may be objectively accessible, yet if students are unaware of its existence, unfamiliar with participation channels, or do not perceive themselves as eligible, the opportunity effectively does not exist for them. Therefore, promoting the occurrence of volunteering behaviors requires not only expanding the supply of opportunities but also attending to how these opportunities are perceived and understood by students. The effectiveness of information dissemination, the accessibility of channels, and the manner in which opportunities are presented all shape students’ subjective appraisal of opportunities and, consequently, their participation decisions (Mato-Santiso et al., 2023).

**Figure 1:**
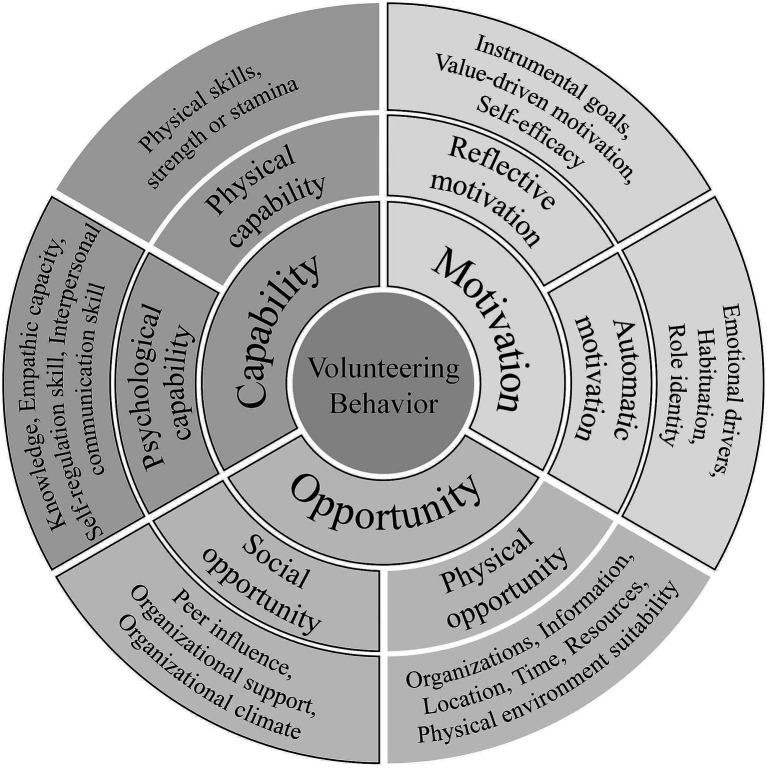
A COM-B–based conceptual framework of university student volunteering behavior.

**Figure 2:**
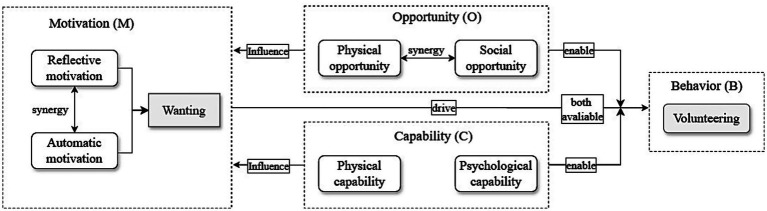
The occurrence mechanism of volunteering behavior from the capability-opportunity-motivation perspective.

Regarding the motivation component, while we emphasize “wanting” as the core driver for behavior, this does not imply that all motivations are equally important or should be cultivated in the same manner. Volunteering motivation is characterized by considerable diversity and individual variability. Different students may participate for entirely different reasons: some seek skill development, others pursue social belonging, some enact altruistic values, and still others simply enjoy the pleasure of helping. This diversity suggests that effective volunteering behavior promotion requires motivational analysis and matching, identifying the predominant motivation types among different student groups and designing differentiated strategies accordingly. At the same time, careful consideration is needed in designing incentive mechanisms. Although extrinsic incentives can increase participation rates in the short term, over-reliance on such incentives may produce a crowding-out effect (Frey and Jegen, 2001), undermining pre-existing intrinsic motivation. We therefore recommend that incentive design place greater emphasis on intrinsic rewards. In the long run, motivations closely tied to identity and intrinsic values are more likely to sustain stable and enduring patterns of volunteer engagement (see Figures 1, 2).

## 6 Conclusion

We introduce the COM-B model to the field of volunteering research, using university student volunteering as an illustrative case to articulate Capability, Opportunity, and Motivation, three conditions required for volunteering behavior to occur. We argue that volunteering emerges from interaction and confluence of these three components at a given moment. Within the Capability component, psychological capability, encompassing volunteering knowledge, empathic capacity, self-regulation skills, and interpersonal communication skills, constitutes the critical determinant for university student volunteering, while physical capability deficits resulting from unhealthy lifestyle habits may serve as hidden barriers; Within the Opportunity component, given that most contemporary universities already provide basic physical opportunity, social opportunity demonstrates greater predictive role. Organizational support, in particular, has emerged as a focus in volunteering research over recent years and plays a pivotal role in facilitating volunteering behavior. Notably, the objective existence of opportunities does not equate to students’ subjective perception of them. How to make all available opportunities genuinely perceptible and accessible to students still remains a noteworthy question; Within the Motivation component, we emphasize the interplay between reflective and automatic motivation. Only when these two types converge into sufficiently strong “wanting” can volunteering behavior ultimately occur.

Our primary contribution lies in the theoretical transfer of the COM-B model and offering a more comprehensive analysis of the conditions that generate volunteering behavior. Naturally, this perspective paper has certain limitations. Given its conceptual nature, the perspectives advanced remain theoretical and require empirical validation in the future. In addition, although university student volunteering is used as an illustrative case, the specific manifestations of capability, opportunity, and motivation may vary across universities with different institutional and cultural contexts. Future research should conduct more empirical investigations, particularly exploring how universities can effectively cultivate students’ psychological capabilities, how opportunities can be made truly perceptible to students, and how to facilitate the interaction between reflective and automatic motivation to generate the strongest possible desire to participate, thereby bridging the intention-behavior gap and ultimately fostering a collective culture of volunteering.

## Data Availability

The original contributions presented in the study are included in the article/supplementary material, further inquiries can be directed to the corresponding author.
